# Changing trends in symptomatology, diagnostics, stage and survival of prostate cancer in Northern Finland during a period of 20 years

**DOI:** 10.1186/1477-7819-11-258

**Published:** 2013-10-05

**Authors:** Outi T Kavasmaa, Dimitri B Tyomkin, Aare Mehik, Teija M Parpala, Panu Tonttila, Ilkka Paananen, Pekka Kunelius, Markku H Vaarala, Pasi Ohtonen, Pekka A Hellström

**Affiliations:** 1 Medical Research Center Oulu, Oulu, Finland; 2 Murmansk Central Regional Hospital, Murmansk, Russia; 3 Division of Operative Care, Department of Urology, Oulu University Hospital, PO BOX 21, Oulu FIN-90029 OYS, Finland

**Keywords:** Prostate cancer, Symptoms, Diagnostics, Chronic prostatitis

## Abstract

**Background:**

Prostate cancer is the most common cancer among men in many countries. The aim of the present study was to find out how the symptoms leading to a diagnosis, diagnostic procedures and stages of the disease among prostate cancer patients have changed over a period of 20 years.

**Methods:**

This retrospective chart review consisted of 421 prostate cancer patients whose treatment was started in the years 1982, 1987, 1992, 1997 and 2002 at the Oulu University Hospital. Earlier prostatic disorders, specific urological symptoms, diagnostic procedures, the TNM classification and histological grade were recorded.

**Results:**

The number of symptom-free prostate cancer patients increased over the 20 years, as did the number of men suffering from chronic prostatitis, although the latter increase was not statistically significant. A drop in the number of clinical T4 cases and increase of clinical T1 and clinical T2 cases was recorded but no clear change in the histological distribution occurred. The 5-year prostate cancer-specific survival improved significantly over the 20 years. The urologist was found to be the person who was contacted first most often.

**Conclusions:**

Our data indicate that the number of prostate cancer patients has increased hugely over the period from 1982 to 2002 and although the clinical T stage has moved towards earlier stages, the proportion of well differentiated cancers remains low, so that most patients have clinically significant cancer with the need of some form of therapy. Further, prostate cancer-specific survival improved significantly over the period.

## Background

Prostate cancer incidence is continuously increasing among men, a trend that can be explained mostly by better diagnostic procedures and the increase in life expectancy for men [1-4]. Despite the huge efforts aimed at prostate cancer research, the prostate-specific antigen (PSA) still remains the main indicator for asymptomatic or mildly symptomatic prostate cancer detection [5]. The aim of the current study was to evaluate how the symptoms leading to prostate cancer diagnosis and the diagnostic procedures used have evolved in Northern Finland over a period of 20 years and what effects these changes have brought with them. This period was selected mainly to evaluate the impact of PSA testing on these diagnostic and clinical aspects, as the early years reflect the pre-PSA era and the PSA test has been available in Finland since 1992.

## Methods

The investigation was retrospective and was based on the review of charts from prostate cancer patients at Oulu University Hospital, whose treatment was started in the years 1982, 1987, 1992, 1997 and 2002. The reference area was served by one university hospital and four central hospitals which have employed at least one urologist since 1982. The total area comprised 724,600 inhabitants, of whom 378,700 live in the primary area of responsibility of the university hospital; these figures have remained quite stable since 1982. Most of the treatments provided in this reference area were not initiated at the university hospital but at the central and regional hospitals. Only patients who were thought to be candidates for radical therapy were referred directly to the university hospital for evaluation. The register of the hospital was searched for patients treated for prostate cancer in the urological department or those who visited the outpatient department in the years in question. This search yielded a total of 421 patients. The research plan was approved by the Ethics Council of the Oulu University Hospital.

### Patient chart review

The following data were collected during patient chart review: all data on the patients’ diagnoses from the patient charts, focusing on earlier prostatic disorders (such as prostatitis and hyperplasia), specific urological symptoms before the first visit to the hospital, such as sexually transmitted diseases, urinary tract infections, dysuria of any kind, haematuria, pain/discomfort and haematospermia, and laboratory results obtained before or at the first visit to the hospital (serum acid phosphatase concentration, serum prostatic acid phosphatase concentration, serum PSA concentration, alkaline phosphatase concentration, haemoglobin value and creatinine value). Other notable symptoms such as bone pain, pathological fractures, anaemia, paraplegia, urinary retention and uraemia were also recorded. Diagnostic procedures performed outside the hospital (such as digital rectal examination (DRE), cytology, biopsy, serum acid phosphatase concentration, serum prostatic acid phosphatase concentration, serum PSA concentration, alkaline phosphatase concentration values) and at Oulu University Hospital (laboratory tests, transrectal ultrasonography (TRUS), cytology, biopsy, bone scan, X-ray, computed tomography (CT), magnetic resonance imaging (MRI), diagnostic transurethral resection of prostate (TURP), date of diagnosis, urinary flow rate and volume of residual urine) were ascertained separately. Symptoms, history of urological diseases and diagnostic findings in the laboratory and radiological examinations were encoded as ‘yes/positive’ or ‘no/negative’. Group ‘yes/positive’ contains cases with the stated specific symptoms or history of urological diseases, and cases with finding supporting prostate cancer diagnosis in the laboratory or radiological examinations. All other cases were break down on ‘no/negative’. We were also interested in the status of the first person to be contacted. The TNM classification [6] and histological findings were taken as part of the diagnosis. The TNM classification took the form of a pathological estimate if the patient had undergone radical prostatectomy, and in other cases it was just a clinical estimate. A histological grading into three World Health Organization classes (well, moderately and poorly differentiated) was used throughout almost the entire period concerned, except in 2002, when all patients were graded according to the Gleason classification. These latter findings were therefore transformed on the assumption that Gleason scores 2 to 4 are identical to well differentiated adenocarcinoma of the prostate, 5 to 7 to moderately differentiated, and 8 to 10 to poorly differentiated.

### Statistical analyses

Data were analysed using the SPSS program (SPSS Inc., Chicago, IL, USA). Comparison between treatment years was done for categorical data using the Chi-Square linear-by-linear association test. Continuous variables were analysed by analysis of variance (ANOVA) or by the Kruskal-Wallis non-parametric test. Two-tailed *P* values are reported. Prostate-cancer-specific survival rates were calculated using the Kaplan-Meier method, and the statistical significance between groups was analysed using the log-rank test.

## Results

There was a trend toward diagnosis at younger age during the period. Mean age of prostate cancer patients whose treatment was started in the years 1982, 1987, 1992, 1997 and 2002 was 70.5 years (range 54–87 years), 70.8 years (49–88 years), 71.2 years (54–88 years), 69.6 years (48–91 years) and 67 years (40–91 years), respectively. The proportion of patients contacting a urologist directly increased, as did the proportion contacting a general practitioner (GP) (Table 1). There was some change in the symptoms that the patients had described before the diagnosis of prostate cancer (Table 2), with the proportion with dysuria or urinary retention of any kind decreasing during the given period (*P*=0.003 for dysuria and <0.001 for urinary retention). Very few patients presented with bone pain. Symptoms and signs such as haematuria, pain/discomfort and haematospermia, paraplegia, pathological fractures and anaemia were also investigated, but the prevalence of these remained stable over the 20 years (data not shown).

**Table 1:** The first person to be contacted leading to a prostate cancer diagnosis

**First contact person**	**1982**	**1987**	**1992**	**1997**	**2002**	**Total**
**GP**						
*n* (%)	20 (48.8)	18 (46.2)	28 (53.8)	75 (64.1)	87 (55.4)	228 (56.2)
**Specialist/surgeon**						
*n* (%)	16 (39.0)	18 (46.2)	18 (34.6)	29 (24.8)	29 (12.1)	100 (24.6)
**Urologist**						
*n* (%)	3 (7.3)	2 (5.1)	5 (9.6)	11 (9.4)	38 (24.2)	59 (14.5)
**Other**						
*n* (%)	2 (4.9)	1 (2.6)	1 (1.9)	2 (1.7)	13 (8.3)	19 (4.7)

**Table 2:** Symptoms before the diagnosis of prostate cancer

**Symptoms**	**1982**	**1987**	**1992**	**1997**	**2002**	**Total**
**Dysuria**						
Yes *n* (%)	29 (65.9)	28 (65.1)	31 (59.6)	67 (55.8)	75 (46.3)	230 (54.6)
No *n* (%)	15 (34.1)	15 (34.9)	21 (40.4)	53 (44.2)	87 (53.7)	191 (45.4)
**Bone pain**						
Yes *n* (%)	4 (9.1)	1 (2.3)	3 (5.8)	5 (4.2)	3 (1.9)	16 (3.8)
No *n* (%)	40 (90.9)	42 (97.7)	49 (94.2)	115 (95.8)	159 (98.1)	405 (96.2)
**Urinary retention**						
Yes *n* (%)	11 (25.0)	9 (20.9)	13 (25)	6 (5)	10 (6.2)	49 (11.6)
No *n* (%)	33 (75)	34 (79.1)	39 (75)	114 (95)	152 (93.8)	372 (88.4)

The number of patients with the history of urinary tract infection, prostatitis/chronic pelvic pain and prostatic hyperplasia is shown in Table 3. The number of patients with the history of benign prostatic hyperplasia increased but not significantly (*P*=0.9). The number of patients with sexually transmitted diseases was very low as there was only one patient in 1987 and in 1992 with the known history of sexually transmitted diseases.

**Table 3:** History of specific urological symptoms before the diagnosis of prostate cancer

**Symptom**	**1982**	**1987**	**1992**	**1997**	**2002**	**Total**
**Urinary tract infection**						
Yes *n* (%)	4 (9.1)	9 (20.9)	5 (9.6)	12 (10.0)	11 (6.8)	41 (9.7)
No *n* (%)	40 (90.9)	34 (79.1)	47 (90.4)	108 (90.0)	151 (93.2)	380 (90.3)
**Prostatitis/chronic pelvic pain syndrome**						
Yes *n* (%)	3 (6.8)	1 (2.3)	5 (9.6)	15 (12.5)	23 (14.2)	47 (11.2)
No *n* (%)	41 (93.2)	42 (97.7)	47 (90.4)	105 (87.5)	139 (85.8)	374 (88.8)
**Benign prostatic hyperplasia**						
Yes *n* (%)	13 (29.5)	18 (41.9)	30 (57.7)	56 (46.7)	61 (37.7)	178 (42.3)
No *n* (%)	31 (70.5)	25 (58.1)	22 (42.3)	64 (53.3)	101 (62.3)	243 (57.7)

**Figure 1:**
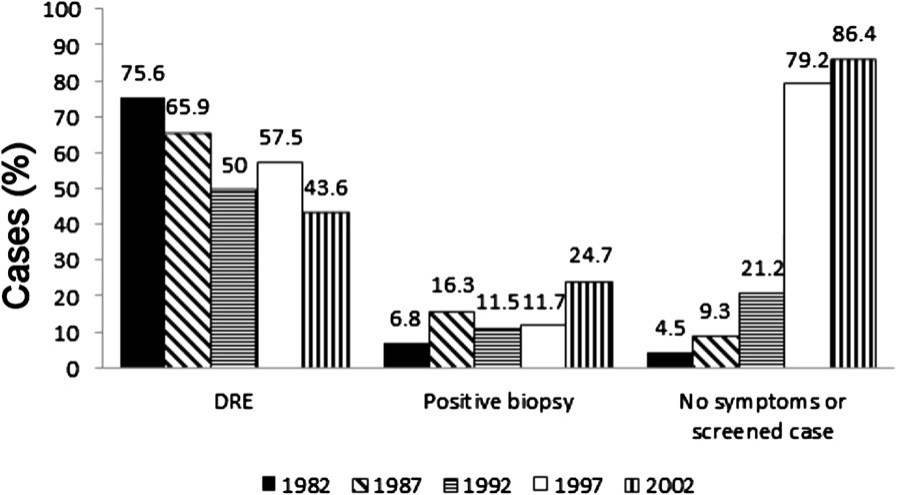
**Diagnostic procedures performed outside the hospital.** DRE: digital rectal examination. No symptoms or screened case: diagnostic examinations performed due to elevated serum acid phosphatase concentration, serum prostatic acid phosphatase concentration or prostate-specific antigen concentration. The numbers above the bars indicate the percentage of patients with specified diagnostic procedures among prostate cancer patients for each respective year.

Diagnostic procedures performed outside the hospital that led to a suspicion or diagnosis of prostate cancer are detailed in Figure 1. The suspicion of prostate cancer had been aroused by a DRE in 33 (75%) out of the 44 patients diagnosed in 1982, but in 70 (43.2%) of the total of 162 patients in 2002. The rest of the patients had no indication of a DRE in their referral, so it is not known whether they were examined or not. The proportion of positive prostate biopsies taken outside the hospital increased from 6.8% in 1982 to 24.7% in 2002, and the proportion of patients with no symptoms and/or screened cases with elevated laboratory values increased from 4.5% to 86.4%. The number (the percentage of cases diagnosed each year) of asymptomatic patients with elevated PSA value was nine (17.3%), 93 (77.5%) and 140 (86.4%) in 1992, 1997 and 2002, respectively (*P* <0.001).

Since prostatic cytology was replaced by histology as a diagnostic method in the course of the period concerned, a cytological sample was taken from only one (0.6%) patient in 2002, while 24 (54.5%) patients were examined in this manner in 1982. Conversely, where only one (2.3%) patient underwent TRUS in 1982, the figure was 25 (58.1%) in 1987, 39 (75%) in 1992, 106 (88.3%) in 1997 and 132(81.5%) in 2002. The use of X-ray examinations for the diagnosis of prostate cancer decreased over the same period, and CT scans and MRI were very seldom used. The number of diagnostic TURPs decreased from 15 (34.1%) out of a total of 44 patients in 1982 to 13 (8%) out of 162 patients in 2002.

**Figure 2:**
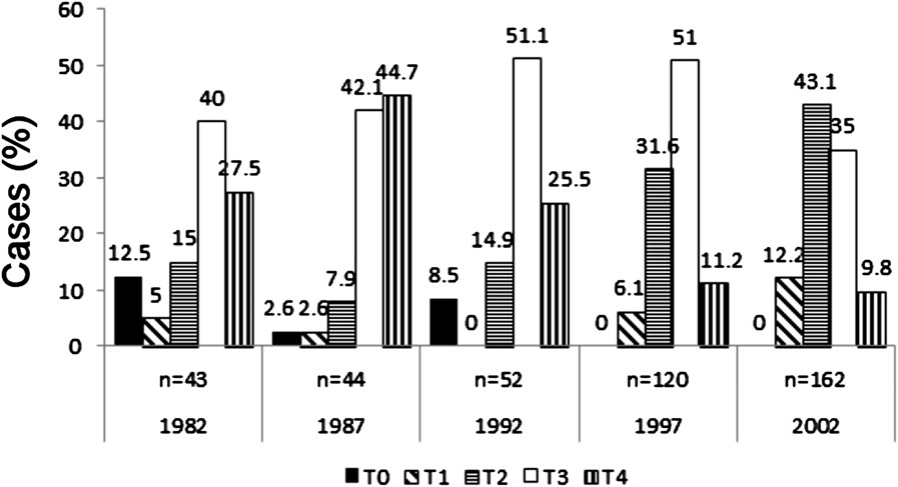
**Clinical T classification (TNM classification) of prostate cancer patients diagnosed throughout the period.** The numbers above the bars indicate the percentage of patients with specified clinical T class among prostate cancer patients for each respective year. Number of diagnosed cases each year is shown (*n*) above the year of diagnosis.

The distribution of clinical T classifications (TNM classification) is presented in Figure 2. There was a clear decrease in the number of clinical T4 cases, whereas clinical T1 and clinical T2 cases increased significantly during the period (*P*= 0.001).

**Figure 3:**
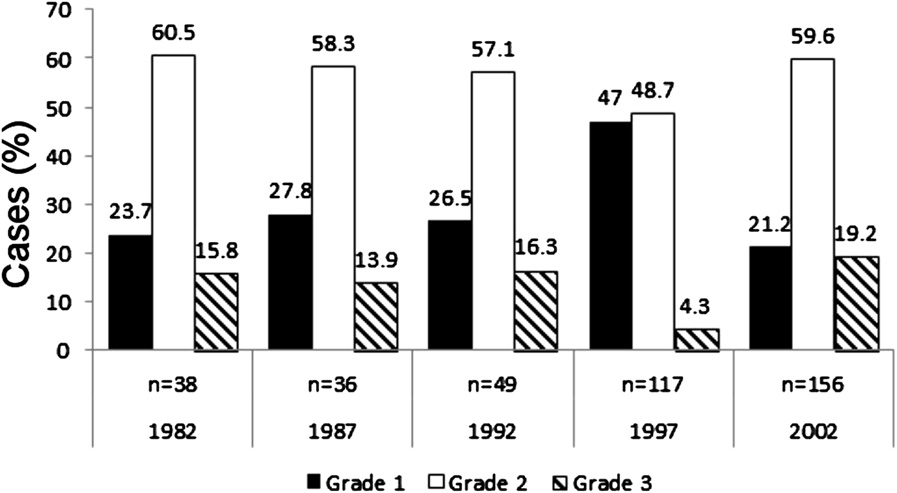
**Histological grade of prostate cancer patient diagnosed throughout the period.** The numbers above the bars indicate the percentage of patients with specified tumour grade among prostate cancer patients at respective year. Number of diagnosed cases each year with grade data available is shown (*n*) above the year of diagnosis.

The trend in histological grading is illustrated in Figure 3. Moderately differentiated cancers were always the most common, while the proportion of well differentiated cancers was highest in 1997.

**Figure 4:**
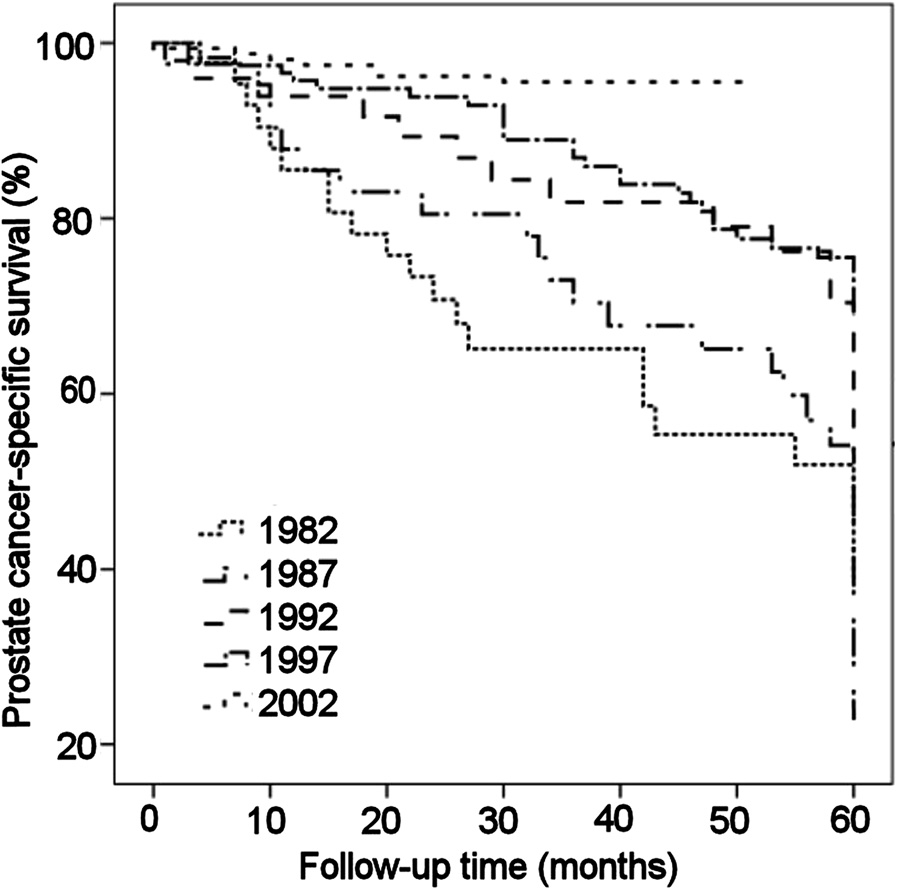
**The 5-year prostate cancer-specific survival of prostate cancer patients with different treatment start year.** The prostate-cancer-specific survival improved significantly (*P* <0.001) depending on the treatment start year.

The 5-year prostate-cancer-specific survival improved during the period (Figure 4, *P* <0.001). Half of the patients and only 4.4% of the patients whose treatment was started in 1982 and 2002, respectively, died of prostate cancer within 5 years.

## Discussion

In this retrospective chart review, we evaluated the symptoms and diagnostic procedures leading to prostate cancer diagnosis in Northern Finland over a period of 20 years. The results point to a clear change in the diagnosis and symptoms of prostate cancer patients over the period. Where 75.6% of the patients in 1982 were diagnosed or a suspicion of prostate cancer was aroused on the basis of DRE, the most frequent finding leading to prostate cancer diagnosis in 1997 was already the PSA test (elevated value in 77.5%). It also appears that GPs may not have performed DRE on all patients. The question therefore arises: do we need DRE at all anymore? Philip et al. [7], who studied whether a DRE is necessary for the diagnosis and clinical staging of early prostate cancer, found no correlation between DRE, biopsy findings and pathological staging, and decided that a DRE may not be essential for patients with a PSA level of 2.5-10 ng/mL. However, a recent report clearly indicates the benefit of DRE in the detection of high risk prostate cancer among men with low PSA [8]. Currently, DRE is essential for the diagnostic procedures associated with prostate cancer [5] despite the fact that prostate cancer diagnostic procedures are due to PSA screening in most cases [9]. DRE, PSA, TRUS and biopsy are the methods that are recommended [5]. A bone scan is also an important diagnostic tool, but should only be used for cases with a high risk of bone metastases [5].

The proportion of patients with dysuria of any kind diminished from 65.9% to 46.3% during the period (Table 2). These results may be explained mostly by that fact that cancers were detected earlier, so such symptoms did not have time to appear. Barrett and Hamilton [10], in their study of the symptoms of prostate cancer, noted that 70% of the prostate cancer patients in Devon, UK, between the years of 1998 and 2002 had lower urinary tract symptoms (LUTS) and that PSA testing had been performed by a GP in 60% of these cases; in contrast, 10% had undergone their first PSA test performed by a urologist. Only 18% of the asymptomatic cancers had been diagnosed by PSA screening. After all, many patients still suffer from overt symptoms, sometimes because of benign prostatic hyperplasia (BPH), which is associated with LUTS. BPH is common among men generally and is also found in many prostate cancer patients, even though it is not considered a premalignant lesion or precursor of carcinoma [11]. Our results indicate that urologists are playing an increasing role as the first people contacted by prostate cancer patients in Northern Finland, but that GPs are still most commonly contacted first. These changes in the availability of healthcare providers partly explain the differences of the first contact person presented in Table 1. In 2002 there were more urologists working in the private sector, meaning that men willing to pay for the examinations were able to contact a urologist directly, while a patient needed a referral from the GP to a urologist during earlier years. Moreover, before PSA testing, the main reason for diagnostic evaluations may have been the symptoms, explaining the high proportion of patients first contacting a specialist/surgeon (Table 1).

Prostatitis and its relationship to prostate cancer have been investigated quite intensively, without definitive conclusions. Some authors have reported a positive association between prostatitis and prostate cancer [12-14], and it has been proposed that inflammation may even lead to prostate cancer [15]. We found here that prostatitis increased among prostate cancer patients, being present in 6.8% of cases in 1982, only 2.3% in 1987, but as many as 14.2% in 2002. However, this result was not statistically significant due to the small number of patients. The rise might be explained by the fact that prostatitis has become better known and diagnosed. Mehik and colleagues [16] showed that the occurrence of prostatitis symptoms in men living in Northern Finland is higher than that reported in many other parts of the world, as the prevalence figure of 14.2% is well above the reported range of 6-10%. They suggest that one cause may be the cold climate, and estimate that the prevalence might be even higher, as there were a considerable number of men who were uncertain about their symptoms. It is quite interesting, however, that the prevalence is exactly the same as the proportion of prostate cancer patients having prostatitis in 2002 in the present survey. This means that prostatitis seems to be no more common among prostate cancer patients than in the population in general. It should also be noted that acute prostatitis essentially raises the PSA level in serum, which can be lowered again by treatments such as anti-inflammatory medication and antibiotics [14,17,18]. This low PSA after treatment does not mean, however, that the individual does not have prostate cancer. Thompson et al. noted a 15.2% prevalence of prostate cancer in men with normal DRE findings and PSA levels of <4.0 ng/mL [19].

The number of prostate cancer patients has increased hugely over the 20 years in question. At the same time, diagnostic procedures and the characteristics of disease were found to have changed, as can be seen in the present findings. Analysis of the clinical T classifications (Figure 2) on a year-by-year basis shows that the dominant classes up to 1992 were cT3 and cT4, whereas cT2 and cT3 were clearly dominant in 1997 and cT2 alone in 2002. cT1 tumours increased between 1997 and 2002, and a similar effect has been noted in a Swedish study, where Varenhorst and colleagues found that the proportion of cT1c tumours increased from 14% to 28% between 1998 and 2002 [20]. All of these changes are probably attributable to the PSA test and an increase in general knowledge of the disease [21]. The increase in the proportion of early stage tumors is the most likely explanation for the significant improvement of prostate-cancer-specific survival detected (Figure 4).

Some interesting findings regarding histological changes in prostate cancer patients have also been reported. Comparing the years 1991 and 2001 in a Scottish series [22], the proportion of patients with Gleason score 5 to 7 cancers was found to have increased, while that of Gleason score 2 to 4 cases decreased. A similar result was obtained in New Mexico, where Gilliand et al. [23] noted that the proportions of Gleason score 5, 6 and 7 tumours increased with time, while those of well differentiated tumours (2, 3 and 4) and poorly differentiated tumours (8, 9 and10) decreased. These results do not support the notion that insignificant, low-grade cancers may have increased along with better diagnostic procedures and earlier diagnosis. It was also the case in the present study that poorly and moderately differentiated cancers were quite common in 2002 (21.2% and 59.6%, respectively) and that no clear change relative to the era before PSA could be seen. There is a bias in this part of our study, however, in that our histological results are partly based on biopsies and partly on prostatectomies, with the results based on biopsy probably not being as reliable.

The present study has several limitations. In a retrospective chart review the availability and quality of the data are limited. In patient charts, specific symptoms may be recorded if they are present, but the absence of recorded sign or symptom does not exclude it which is possible to discriminate in prospective setting. Further, although prostate cancer cases were extensively searched based on diagnosis numbers and codes, some cases may be missed due to wrong encoding, which may reflect in the results. The small number of patients especially during early years of the study may limit the ability to detect significant trends.

## Conclusions

There was a clear change in the diagnosis and symptoms of prostate cancer patients in Northern Finland over the 20-year period examined here. PSA testing is likely to be the main explanatory factor for this change. The clinical T class results suggest that prostate cancer may have become a milder disease, but the histological grading does not support such a conclusion. However, 5-year prostate-cancer-specific survival improved significantly over the 20 years under examination.

## Competing interests

The authors declared that they have no competing interests.

## Authors’ contributions

OTK and DBT did the chart review. AM, TMP, PT, IP, PK and PAH participated in the design of the study and drafted the manuscript. MHV and PO performed the statistical analysis. All authors read and approved the final manuscript.
